# LAP degradation product reflects plasma kallikrein-dependent TGF-β activation in patients with hepatic fibrosis

**DOI:** 10.1186/2193-1801-3-221

**Published:** 2014-05-01

**Authors:** Mitsuko Hara, Akiko Kirita, Wakako Kondo, Tomokazu Matsuura, Keisuke Nagatsuma, Naoshi Dohmae, Shinji Ogawa, Shinobu Imajoh-Ohmi, Scott L Friedman, Daniel B Rifkin, Soichi Kojima

**Affiliations:** Micro-signaling Regulation Technology Unit, RIKEN Center for Life Science Technologies, 2-1 Hirosawa, Wako, Saitama, 351-0918 Japan; Department of Laboratory Medicine, The Jikei University School of Medicine, Minato-ku, Tokyo, 105-0003 Japan; Division of Gastroenterology and Hepatology, Department of Internal Medicine, The Jikei University School of Medicine, Minato-ku, Tokyo, 105-0003 Japan; Biomolecular Characterization Team, Chemical Biology Core Facility, Chemical Biology Department, RIKEN Advanced Science Institute, Wako, Saitama, 351-0918 Japan; St. Louis Laboratories, Pfizer Worldwide Research & Development, Chesterfield, MO 63166 U.S.A; Institute of Medical Science, University of Tokyo, Minato-ku, Tokyo, 108-8639 Japan; Division of Liver Diseases, Icahn School of Medicine at Mount Sinai, New York, NY 10029 U.S.A; Department of Cell Biology, New York University School of Medicine, New York, NY 10016 U.S.A

**Keywords:** Biomarker, Hepatic stellate cells, Liver fibrosis, Plasma kallikrein (PLK), TGF-β activation

## Abstract

Byproducts of cytokine activation are sometimes useful as surrogate biomarkers for monitoring cytokine generation in patients. Transforming growth factor (TGF)-β plays a pivotal role in pathogenesis of hepatic fibrosis. TGF-β is produced as part of an inactive latent complex, in which the cytokine is trapped by its propeptide, the latency-associated protein (LAP). Therefore, to exert its biological activity, TGF-β must be released from the latent complex. Several proteases activate latent TGF-β by cutting LAP. We previously reported that Camostat Mesilate, a broad spectrum protease inhibitor, which is especially potent at inhibiting plasma kallikrein (PLK), prevented liver fibrosis in the porcine serum-induced liver fibrosis model in rats. We suggested that PLK may work as an activator of latent TGF-β during the pathogenesis of liver diseases in the animal models. However, it remained to be elucidated whether this activation mechanism also functions in fibrotic liver in patients.

Here, we report that PLK cleaves LAP between R^58^ and L^59^ residues. We have produced monoclonal antibodies against two degradation products of LAP (LAP-DP) by PLK, and we have used these specific antibodies to immunostain LAP-DP in liver tissues from both fibrotic animals and patients.

The N-terminal side LAP-DP ending at R^58^ (R^58^ LAP-DP) was detected in liver tissues, while the C-terminal side LAP-DP beginning at L^59^ (L^59^ LAP-DP) was not detectable. The R^58^ LAP-DP was seen mostly in α-smooth muscle actin-positive activated stellate cells.

These data suggest for the first time that the occurrence of a PLK-dependent TGF-β activation reaction in patients and indicates that the LAP-DP may be useful as a surrogate marker reflecting PLK-dependent TGF-β activation in fibrotic liver both in animal models and in patients.

**Electronic supplementary material:**

The online version of this article (doi:10.1186/2193-1801-3-221) contains supplementary material, which is available to authorized users.

## Background

**Figure 1:**
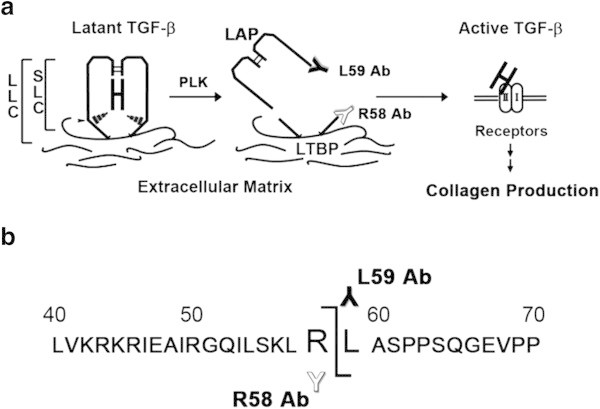
**Determination of the LAP cleavage site produced during PLK-dependent TGF-β activation. (a)** Schematic depiction of latent TGF-β1 activation by PLK. PLK cleaves LAP between R^58^ and L^59^ residues, which may destroy the interaction between LAP and active TGF-β1 molecule, releasing active TGF-β1 from the latent complex. LLC; large latent complex, SLC; small latent complex, LTBP; latent TGF-β binding protein. **(b)** The PLK cleavage site was determined by amino acid sequencing of LAP-DP isolated after SDS-PAGE. The amino acid sequences around the PLK cleavage site are illustrated. Antibodies (Ab), which specifically recognize cutting edges of LAP-DPs, were produced. White “Y” represents R58 antibody recognizing the N-terminal side R^58^ LAP-DP, whereas black “Y” represents L59 antibody recognizing the C-terminal side L^59^ LAP-DP.

Liver fibrosis is characterized as the pathological deposition of excessive extracellular matrix (ECM), which finally causes hepatic dysfunction (Bataller and Brenner 2005). A key driver of fibrosis is the 25 kD homodimeric cytokine, transforming growth factor (TGF)-β (Dooley and ten Dijke 2012). TGF-β is produced as a latent complex; therefore, an essential step for controlling TGF-β activity is its activation, a process in which biologically active TGF-β is released from the latent complex (Figure 1a) (Dabovic and Rifkin 2008).

TGF-β1 is produced as a 390 amino acid precursor protein consisting of a signal peptide of 29 amino acids, an N-terminal pro-region called latency-associated protein (LAP), and a C-terminal region that becomes the active TGF-β1 molecule, and each region dimerized through S-S bonds. After processing by cleavage at R^278^-A^279^ by a furin-like protease, the LAP non-covalently binds the mature TGF-β1, forming small latent complex (SLC) and preventing active TGF-β1 from binding its cognate receptors (Figure 1a). The active TGF-β1 and the LAP homodimer are 25 kD and 75 kD, respectively. The SLC is S-S bonded to another gene product, the latent TGF-β binding protein (LTBP), via C^33^ residues, forming the large latent complex (LLC). This complex can be sequestered in the ECM (Figure 1a) (Breitkopf et al. 2001). LTBP is a member of an ECM protein family, fibrillin (Zilberberg et al. 2012).

Several molecules are known to activate TGF-β1 in animal models. These include integrins (Nishimura 2009; Henderson et al. 2013), thrombospondin (Ribeiro et al. 1999), and proteases, such as matrix metalloproteinases (MMPs) (Yu and Stamenkovic 2000) and serine proteases (Jenkins 2008). Lyons R.M. *et al*. first reported that plasmin digests LAP and activates TGF-β1 *in vitro* (Lyons et al. 1990). Using a protease inhibitor, Camostat Mesilate, we previously demonstrated that plasma kallikrein (PLK) is involved in the TGF-β1 activation associated with liver fibrosis and impaired liver regeneration in animal models (Okuno et al. 2001; Akita et al. 2002). However, it remained to be elucidated whether PLK-dependent TGF-β1 activation also occurs during the pathogenesis of liver fibrosis in patients.

In this paper, we describe successful experiments aimed at generating specific antibodies against the two degradation products of LAP (LAP-DP) produced after PLK digestion, and the use of these antibodies to stain the LAP-DP in patient livers, thereby providing evidence of PLK-dependent TGF-β1 activation in human hepatic fibrosis. The results demonstrate the potential utility of the LAP-DP as a surrogate marker for PLK-dependent activation of TGF-β1 in the liver.

## Results

### Identification of LAP cleavage sites during proteolytic activation of latent TGF-β1

PLK primarily cleaved recombinant human LAP β1 (rhLAP β1) between R^58^ and L^59^ residues (Figure 1b). Further incubation resulted in cleavage between R^267^and A^268^ residues.

### Preparation of specific antibodies that recognize LAP neo-epitopes formed by PLK during TGF-β1 activation

**Figure 2:**
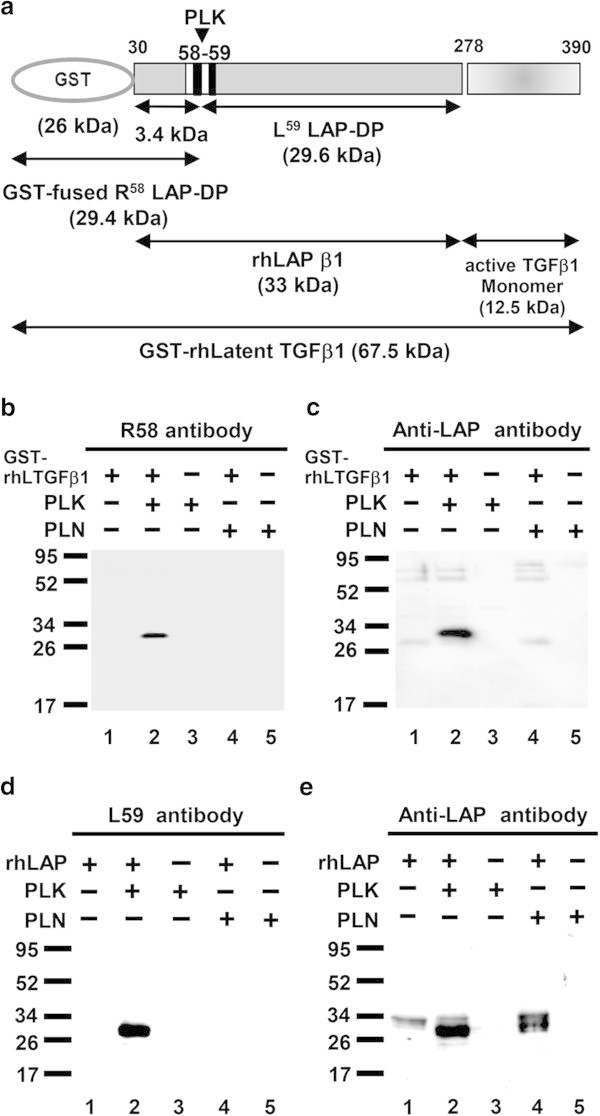
**Specificity of R58 and L59 antibodies. (a)** Schematic structure of a GST- rhLTGF-β1 protein generated in bacteria and a commercial rhLAP β1 generated in insect cells. The PLK cleavage site is indicated by arrowhead. **(b-e)** GST-LTGF-β1/rhLAP β1was incubated with/without PLK or PLN for 45 min at 37°C, and thereafter equal amounts of samples containing either 50 ng GST-rhLTGF-β1 or 500 ng rhLAP β1 were subjected to each lane in SDS-PAGE under reducing conditions followed by Western blot analyses using R58/L59 antibodies **(b, d, respectively)**. Membranes used in panels b and d were reprobed with monoclonal **(c)** or polyclonal **(e)** anti-LAP antibodies. *Lanes 1*, intact GST-rhLTGF-β1 or rhLAP-β1; *lanes 2*, PLK digested GST-rhLTGF-β1 or rhLAP-β1; *lanes 3*, PLK; *lane 4*, PLN digested GST-rhLTGF-β1 or rhLAP-β1; *lane 5,* PLN. Representative results from three independent experiments with a similar result are shown.

Based on the amino acid sequences of PLK cleavage site, we prepared monoclonal antibodies that recognized the neo-epitopes formed within LAP during PLK-dependent TGF-β1 activation (Figure 1). The antibodies against the neo-C-terminal end of the PLK-cleaved N-terminal side LAP-DP ending at R^58^ (referred as R^58^ LAP-DP) and the neo-N-terminal end of the PLK-cleaved C-terminal side LAP-DP beginning from L^59^ (referred as L^59^ LAP-DP) were named R58 and L59 antibodies, respectively. Figure 2 shows Western blots using Glutathione-*S*-transferase (GST) fused-recombinant human latent TGF-β1 (GST-rhLTGF-β1) or rhLAP β1 (schematic structures are illustrated in panel a) to examine the specificities of R58 and L59 antibodies. The R58 antibody recognized only GST-fused R^58^ LAP-DP around 30 kD (panel b, lane 2), and the L59 antibody recognized the 29 kD of L^59^ LAP-DP (panel d, lane 2). However, both R58 and L59 antibodies did not recognize uncleaved GST-rhLTGF-β1 and rhLAP β1 (panels b and d, lane 1) that were detectable by the monoclonal anti-LAP antibody recognizing N-terminal side LAP-DP (panel c) or the polyclonal anti-LAP antibody recognizing C-terminal side LAP-DP (panel e). Moreover, neither R58 nor L59 antibodies detected LAP-DPs generated by plasmin (PLN) digestion (panels b and d, lane 4) These data demonstrated that the anti-LAP antibodies recognize both LAP and PLK-cleaved LAP-DP, while the R58 and L59 antibodies specifically recognize only the respective LAP-DP. There are three isoforms of pro-TGF-βs. Amino acid sequences between E^47^ and L^59^ residues in LAP are identical between TGF-β1 and TGF-β3 (Additional file 1: Figure S1). Hence, we checked whether R58 antibody can recognize TGF-β3 LAP-DP as well as TGF-β1 LAP. As shown in Additional file 2: Figure S2, R58 antibody recognized PLK-digested TGF-β1 and β3 LAP peptide, but not non-digested and PLN-digested TGF-β1 and β3 LAP peptides (upper and lower lane, respectively) nor TGF-β2 LAP peptide, which does not have P1 site R^58^ residue, irrespective of PLK or PLN digestion (middle lane). These data suggested that R58 and L59 antibodies are promising tools to monitor cleavage of LAP by PLK, in other words, both R^58^ and L^59^ LAP-DPs can be footprints of PLK-dependent TGF-β1 as well as TGF-β3 activation.

### Immunohistochemistry of murine fibrotic liver using R58 antibodies

**Figure 3:**
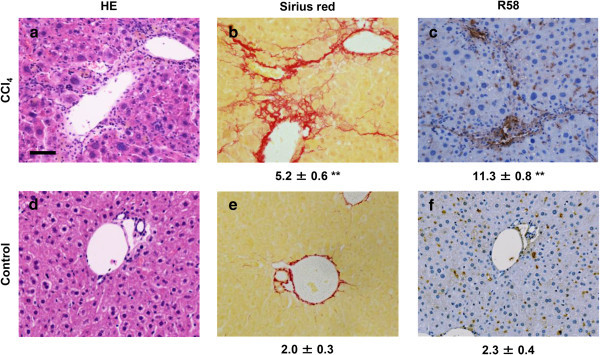
**Immunohistochemistry of R** ^**58**^ **LAP-DP in fibrotic liver tissues in CCl**_**4**_ **-treated mice using R58 antibody.** Liver tissues from CCl_4_-treated and control (olive oil administrated) mice were stained by HE **(a, d)**vand Sirius Red ***(*** **b, e)**, and then immunostained with R58 antibody **(c, f)**. Scale bar = 50 μm. The percentage of fibrotic regions and R58 positive areas were calculated from three fields each from five sections (5 mice) (total 15 fields) and described as average ± SE under the corresponding panels. A total of 16 mice were evaluated and representative results are shown.

**Figure 4:**
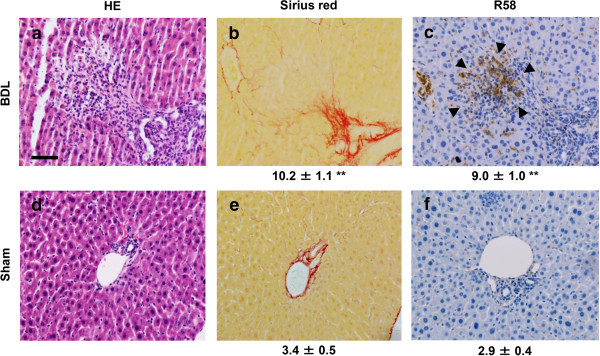
**Immunohistochemistry of R** ^**58**^ **LAP-DP in fibrotic liver tissues in BDL mice using R58 antibody.** Liver tissues from BDL and sham-operated mice were stained by HE **(a, d)** and Sirius Red **(b, e)**, and then immunostained with R58 antibody **(c, f)**. Scale bar = 50 μm. The percentage of fibrotic regions and R58 positive areas were calculated from three fields each from five sections (5 mice) (total 15 fields) and described as average ± SE under the corresponding panels. A total of 16 mice were evaluated and representative results are shown.

**Figure 5:**
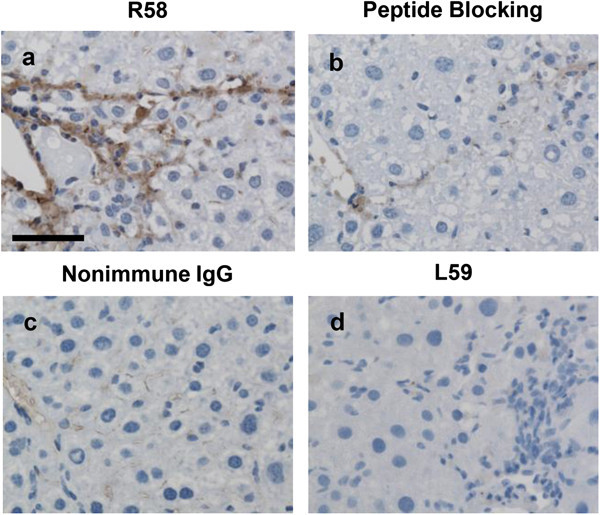
**Control experiments for immunostaining with R58 and L59 antibodies.** Serial liver sections of from CCl_4_-treated mice at 12 weeks were stained with R58 antibody (1 μg/ml) **(a)**, R58 antibody (1 μg/ml) that had been pre-incubated with 10 μg/ml R58 antigen peptide **(b)**, the same concentration of nonimmune mouse IgG **(c)**, and L59 antibody (1 μg/ml) **(d)**. Scale bars = 25 μm. This picture shows the representative result from three independent experiments.

**Figure 6:**
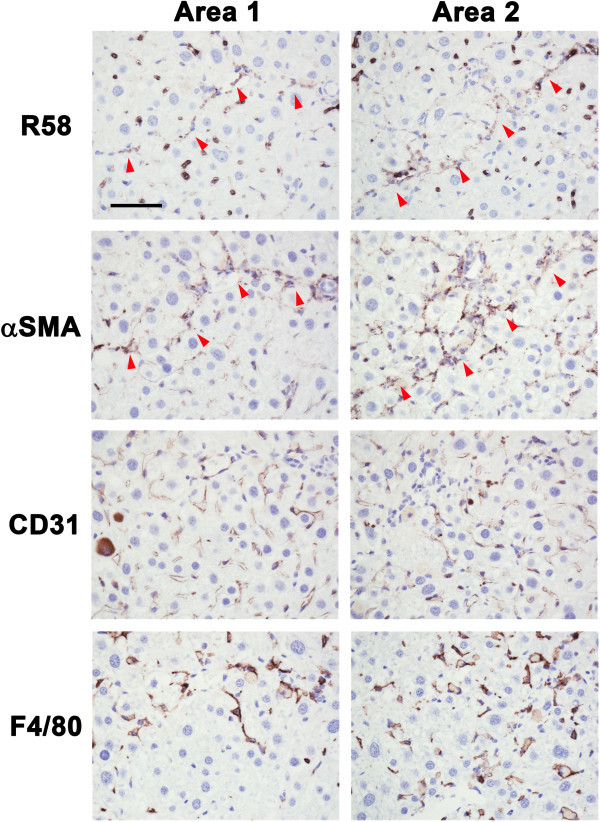
**Identification of R58 positive cells in CCl**_**4**_ **-treated mice liver sections.** Paraffin serial sections were immunostained with, R58 (upper panels), anti-αSMA (second row panels), anti-CD31 (third row panels), and anti-F4/80 (lower panels) antibodies. R58 positive signals were compared with αSMA, CD31, or F4/80 signals in same areas of each sections. The red arrowheads (R58 and αSMA panels) represent respectively both R58 and αSMA positive cells along with fibrotic septa. Scale bar = 50 μm.

**Figure 7:**
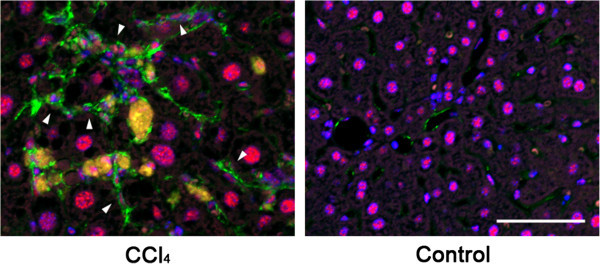
**Detection of phosphorylated Smad3C in activated HSCs in CCl**_**4**_ **-treated mice liver sections.** Phosphorylation and nuclear translocation of Smad3C in activated HSCs were examined by immunofluorescent double staining with anti-pSmad3C (red) and anti-αSMA (green) antibodies. Nuclei of the cells were identified by Hoechst 33258 staining (blue). The pSmad3C positive-activated HSCs were seen only in CCl_4_-treated mice. Scale bar = 50 μm.

Because LAP may be S-S bonded to LTBP present in the ECM, we expected that the R^58^ LAP-DP would remain within fibrotic tissues after cleavage of LAP by PLK. We tested this possibility in fibrotic livers from the carbon tetrachloride (CCl_4_)-treated mice and bile duct ligated (BDL)-mice using R58 antibody. As shown in Figure 3, at 12 weeks after initiating CCl_4_ treatment, CCl_4_-treated mice displayed hepatocellular injury around the lobes (panel a) and bridging fibrosis from central veins to portal areas (panel b). The R^58^ LAP-DP was detectable and increased in CCl_4_-treated mice (panel c), especially along fibrotic areas in CCl_4_-treated mice compared with olive oil treated control mice. Positive area rates were increased 5-folds as written underneath panels c and f. Small positive spots seen in the control section were non-specific staining of mostly erythrocytes (panel f). Similar results were obtained from BDL mice (Figure 4). BDL mice often exhibited granulomatous lesions (panel a), in which fibroblastic cells infiltrated and started ECM production (panel b). The R^58^ LAP-DP was detected in granulomatous lesions prior to Sirius red positivity, namely before collagen accumulation (arrowheads in panel c). Positive areas were increased 3-folds as written underneath panels c and f. Signals from R^58^ LAP-DP was absorbed by pre-incubation of R58 antibody with antigen-peptides, and non-immune mouse IgG failed to yield a signal (Figure 5a-c), indicating that the signals were specific to R^58^ LAP-DP. We were unable to stain the L^59^ LAP-DP with L59 antibody although various antigen unmasking procedures were treated (Figure 5d). In, Figure 6, non-parenchymal regions were recognized by antibody R58 (red arrowheads in upper panels), and mostly overlapped with α-smooth muscle actin (αSMA)-positive activated hepatic stellate cells (HSCs) (red arrowheads in second row panels*)*, but not with CD31-positive liver sinusoidal endothelial cells (third row panels) nor with F4/80-positive Kupffer cells (hepatic macrophages) (lower panels). Activated HSCs in the fibrotic liver were stained strongly with anti-pSmad3C antibody compared to olive oil-treated control mice (arrowheads in Figure 7, left panel), suggesting that TGF-β signaling was provoked. These data suggest that PLK-dependent TGF-β1/3 activation was induced in murine liver fibrosis models and that R^58^ LAP-DP, but not L^59^ LAP-DP, can be a footprint of the generation of active TGF-β1/3 in liver tissue.

### Immunohistochemistry of fibrotic liver tissues from patients using R58 antibody

**Figure 8:**
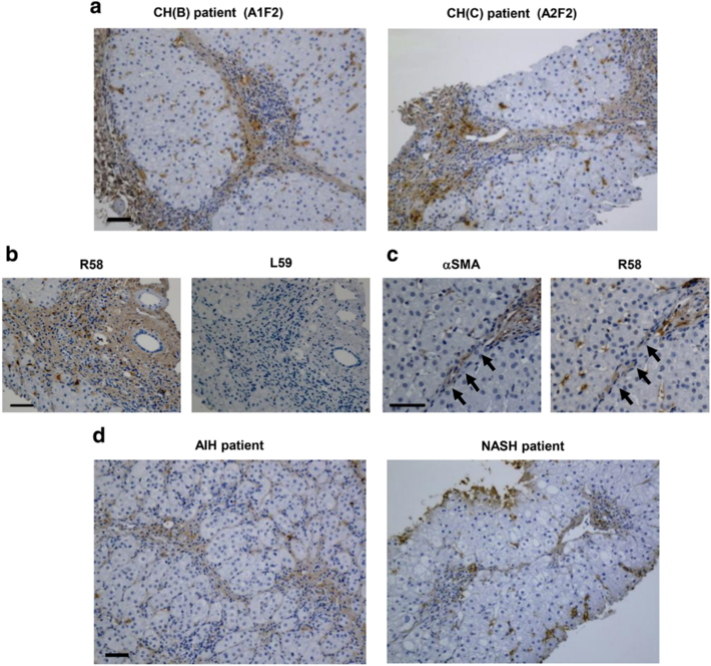
**Immunostaining of biopsied human liver tissues. (a, d)** Liver biopsy samples from four patients (chronic hepatitis B and C virus infection [CH (B) and (C), respectively], AIH with severe fibrosis, and NASH. The hepatitis activity grade and fibrosis score are described in the upper corresponding micrograms. **(b)** Serial sections from CH(C) patient were stained with R58 and L59 antibodies. **(c)** Liver specimens from another CH(B) patient were stained with anti-αSMA, R58 antibodies. αSMA and R58 signal positive cells lined up along fibrous septa (yellow arrows). Scale bars = 50 μm.

Finally, we evaluated human liver sections with the R58 antibody to determine if PLK-dependent TGF-β1/3 activation occurs in clinical liver diseases. Fibrotic liver tissues from patients with viral hepatitis categorized as A1F2 and A2F2 were stained robustly with the R58 antibody (Figure 8a). As with animal models, no signals could be detected by L59 antibody (Figure 8b). In peri-sinusoidal and fibrotic regions around the hepatic lobes, R^58^ LAP-DP was present in non-parenchymal cells, mainly in αSMA-positive HSC (arrows in Figure 8c). R58 signals could be detected along fibrous septa, implying that R^58^ LAP-DP accumulated within the ECM upon TGF-β1/3 activation. A similar staining with the R58 antibody was observed in patients with non-viral hepatitis, such as autoimmune hepatitis (AIH) and non-alcoholic steatohepatitis (NASH) (Figure 8d). These data clearly indicated that PLK-dependent TGF-β1/3 activation is induced during the pathogenesis of liver fibrosis in patients with a range of liver diseases, and that PLK-cleaved R^58^ LAP-DP might serve as a novel surrogate biomarker of TGF-β1/3 activation and its associated fibrosis in patients.

## Discussion

An important step to control biological activity of TGF-β is its activation referred as “TGF-β activation.” Proteolytic activation of latent TGF-β1 was first described in 1988 (Lyons et al. 1988) and was demonstrated in hepatic stellate cells during the pathogenesis of liver fibrosis and/or impaired liver regeneration in animal models (Okuno et al. 2001; Akita et al. 2002). Although we described PLK-dependent TGF-β activation in rodent models of the liver diseases, it remained to be elucidated whether this mechanism is also effective in patients.

Here, we have identified PLK cleavage site within the LAP of latent TGF-β1, and have generated antibodies that specifically recognize the neo C- and N-termini of LAP-DPs (R58 and L59 antibodies, respectively) (Figure 1). These antibodies may be powerful tools to detect the footprints of latent TGF-β1 activation. Notably, we successfully used the antibodies to detect LAP-DP generated during TGF-β1 activation in human samples. For the first time, we demonstrated that the PLK-dependent activation of latent TGF-β1 is associated with human hepatic diseases with the use of the R58 antibody. This technique can be generally applied to measure activation of other cytokines, in which by-products corresponding to the LAP-DP are generated.

The primary cleavage site of LAP by PLK was between R^58^-L^59^ (Figure 1b). Interestingly, the PLK cleavage site is proximal with the putative binding site for thrombospondin (L^54^SKL^57^) (Ribeiro et al. 1999). This suggests that the region containing L^54^SKLRL^59^ is important for maintaining TGF-β1 latency. Consistent with this idea, it was recently revealed that the active TGF-β1 molecule is associated with this region based both on mutational analysis (Walton et al. 2010) and the crystal structure of porcine latent TGF-β1 (Shi et al. 2011). Specifically, this sequence is located near the C-terminal end of α1 helix of LAP and hydrophobic residues containing L^54^, L^57^, and L^59^, which are present along the inner face of the α1 helix, interact with W^308^/W^310^ and with aliphatic side chains of active TGF-β1. The α1 helix is predicted to play a role of a ‘straitjacket’ for active TGF-β1 (Walton et al. 2010). The K^56^ fastens the C-terminal end of the α1 helix in cooperation with Y^74^-A^76^, and moreover associates S^380^ via hydrogen bonding (Shi et al. 2011). The P1 site amino acids (R^58^) are located on the outer face of LAP, so PLK cleavage site should be highly accessible. Proteolytic cleavage within this region is likely to cause a conformational change of the LAP, resulting in a loss of its association with the active TGF-β1 molecule and release of active TGF-β1 molecule from the latent complex.

In Figure 2, we made monoclonal antibodies against both N- and C-terminal side LAP-DP (R^58^/L^59^ LAP-DP) and checked their reaction specificities by western blotting. R58/L59 antibodies detected neither uncleaved LAP nor PLN-cleaved LAP-DP, and specifically recognized only PLK-cleaved LAP-DP, whereas anti-LAP antibodies recognized both uncleaved and PLK-cleaved LAP-DP. In Figure 2c, lane 4, we failed to detect N-terminal side LAP-DP made by PLN digestion with monoclonal anti-LAP antibody that nicely detected N-terminal side LAP-DP made by PLK digestion. We speculate that the epitope recognized by this anti-LAP antibody would be lost by PLN digestion.

Three isoforms of proTGF-β (β1-β3) have been characterized (Massague 1990), and LAP domains of different isoforms share 28-45% identity. TGF-β2 LAP does not have the sequences cleaved by PLK, whereas TGF-β3 LAP has the same sequences as TGF-β1 LAP. Our results indicated that PLK can cleave TGF-β3 LAP, and that their degradation products were detectable by R58 antibody. Because TGF-β3 expression is reported to be very weak in the liver (De Bleser et al. 1997), we think it is likely that the R^58^ LAP-DP we detected is derived mainly from TGF-β1 LAP, but partly from TGF-β3 LAP.

We also successfully detected PLK-cleaved R^58^ LAP-DP using the R58 antibody in fibrotic mice, suggesting that PLK dependent TGF-β1/3 activation was at least involved in murine hepatic fibrosis and that PLK-cleaved LAP-DP might be a marker for liver fibrosis. Our previous study demonstrated that PLN also plays an important role in liver fibrosis in the rat porcine serum model (Okuno et al. 2001), therefore we are now using the same strategy to produce antibodies that recognize PLN-cleaved LAP-DP. Similar to murine models, R^58^ LAP-DP was observed in patients suffering from hepatic diseases. Fibrous septa were obviously stained by the R58 antibody, indicating that R^58^ LAP-DP was generated at the site of the TGF-β1/3 activation and remained there. The intensity of R58 signals did not correlate with the severity of hepatic inflammation and fibrosis, so future studies will assess what drives increased R58 expression in vivo.

In contrast to the R58 antibody, we obtained essentially no positive signals with L59 antibody staining in both rodent and human samples. This suggests that C-terminal side L^59^ LAP-DP might be released after proteolytic digestion. Recently, we established an ELISA using the L59 antibody and successfully detected PLK-cleaved L^59^ LAP-DP generated in in vitro reactions. Currently, we are examining if we can detect L^59^ LAP-DP in plasma from animal models and patients, and if so, what is the clinical relevance of these values in liver diseases.

Integrins are known to activate TGF-β1, and it is reported that several subtypes of integrin, for example, α_v_β6, are related to hepatic fibrosis in both animal models and patients (Patsenker et al. 2008; Henderson and Sheppard 2013; Allison 2012). Because integrins, which are anchored to the cell membrane, stretch the LAP by interacting via the RGD motifs to release active TGF-β1, the LAP DP is not produced (Wipff et al. 2007). Therefore compared to integrins, PLK-cleaved R^58^ LAP-DP will serve as a more direct biomarker for TGF-β1 activation and following liver fibrosis.

Du X et al. showed that MMP-2 is related to renal fibrosis (Du et al. 2012). Several MMPs are also known to activate latent TGF-β1 (Yu and Stamenkovic 2000), although their cleavage sites within LAP have not been determined. Therefore, we are expanding our current method to detect MMP-mediated TGF-β1 activation in the kidney.

## Conclusions

In summary, we demonstrate here 1) PLK cleaves LAP between R58 and L59 residues, 2) PLK-dependent TGF-β activation occurs in human hepatic fibrosis, and 3) PLK-cleaved LAP-DP is the potential surrogate marker for proteolytic TGF-β1/3 activation and in turn fibrogenesis in the liver.

## Methods

### Materials

Recombinant human TGF-β1 LAP (rhLAP β1) and both monoclonal and polyclonal anti-human LAP β1 antibodies (MAB246 and AF246, respectively) were purchased from R&D Systems (Minneapolis, MN). Human PLK was from CalBiochem (San Diego, CA). Anti-αSMA antibody were purchased from DAKO (Glostrup, DK). Anti-CD31 antibody was from dianova GmbH (Hamburg, DE). Anti-F4/80 antibody was from AbD serotec, LLC (Oxford, UK). Anti-pSmad3C polyclonal antibody was a kind gift from Dr. Matsuzaki (Kansai Med. Univ., Japan). GST-rhLTGF-β1 protein was prepared as described follows. A plasmid encoding GST-rhLTGF-β1 was constructed by inserting a human TGF-β1 cDNA into the pGEX-6P-1 vector (GE Healthcare, Buckinghamshire, UK), and protein expressed in *E. coli.* BL21 (Stratagene, La Jolla, CA) and purified using Glutathione Sepharose (GE Healthcare).

### Determination of the cleavage sites within LAP by PLK

To identify the cleavage site in LAP during latent TGF-β1 activation by PLK, rhLAP β1 was incubated with PLK. After digestion, the resultant fragments were separated by SDS-polyacrylamide gel electrophoresis, and the N-terminal sequence of each LAP-DP was determined using a pulsed liquid protein sequencer Precise 494cLC (Hayashi et al. 2008).

### Preparation of R58 and L59 monoclonal antibodies against neo C- and N-termini of LAP-DPs generated by PLK

Murine R58 and L59 monoclonal antibodies were generated against an 8 amino acid peptide, ending at R^58^ and plus a CG linker sequence at its N-terminus [CGGQILSKLR (Figure 1b)] and an 11 amino acid peptide, beginning from L^59^ and plus a GGC linker sequence [LASPPSQGEVPGGC (Figure 1b)]. BALB/c mice purchased from Charles River Laboratories Japan, Inc. (Kanagawa, Japan) were immunized with 50 μg of the antigen peptides. Once an appropriate titer had been achieved, fusion was performed using a protocol adapted from Lane *et al*. (Lane et al. 1986). Positive clones, which reacted to the BSA-conjugated antigen peptide, but not to the terminus-modified antigen peptide (the C-termini were amidated for R58 antibody, while the N termini were acetylated for L59 antibody production), were selected. The antibodies were purified through the Protein G column (GE Healthcare).

### SDS-PAGE and Western blot analysis

GST-rhLTGF-β1 as well as rhLAP β1 were digested by co- incubation with PLK or PLK at 37°C for 45 min. Thereafter, equal amounts of samples containing either 50 ng of GST-rhLTGF-β1 or 500 ng of rhLAP β1 were subjected to each lane in SDS-PAGE under reducing conditions, followed by transfer onto PVDF membrane (Millipore, Bedford, MA). Western blot analyses were performed using either monoclonal R58 and L59 antibodies (2 μg/ml) plus HRP-conjugated anti-mouse antibodies (1:5,000) (Jackson Immuno Research Laboratories, Inc., West Grove, PA) and reprobed either with monoclonal or polyclonal anti-LAP antibodies. The bands were visualized by a Western Blotting Substrate Plus purchased from Thermo Scientific (Rockford, IL).

### Animal models

Male C57BL/6 mice were purchased from Japan SLC Inc. (Shizuoka, Japan). All animals were maintained on a 12 hour light/12 hour dark cycle. Food and water were available *ad libitum*. All animal experiments were performed in accordance with protocols approved by the RIKEN Institutional Animal Use and Care Administrative Advisory Committee. For the CCl_4_-induced liver fibrosis model, mice were injected intramuscularly with 50% CCl_4_ (CCl_4_: olive oil = 1:1, 2 ml/kg) twice a week for 12 weeks. Control animals were injected with the same volume of olive oil. Mice were sacrificed one week after the last CCl_4_ injection. For the BDL model, ligation of the common bile duct was performed according to Arias *et al*. (Arias et al. 2003). Sham-operated mice were treated in the same manner except that the bile duct was not ligated. The animals were sacrificed at 14 days after operation.

### Patient samples

The study was performed in accordance with the Declaration of Helsinki and was approved by the Ethics Committee for Biomedical Research of the Jikei University School of Medicine and RIKEN Institute Research Ethics Committee. All patients had signed a written informed consent prior to study. Liver biopsy samples were taken from patients suffering with cirrhotic liver diseases due to infection with types B and/or C hepatitis virus, AIH and NASH.

The stage of fibrosis and the grade of inflammatory activity were classified according to the METAVIR score (Bedossa and Poynard 1996).

### Staining of liver tissue sections

Animal tissue specimens were fixed in 4% paraformaldehyde, and human biopsied samples were fixed in 10% neutral buffered formalin. They were all embedded in paraffin. Sections (4 μm thickness) were stained by Hematoxylin and Eosin (HE), as well as 0.1% picro-sirius red solution for diagnostic purposes. For immunostaining of the LAP-DP, antigens were retrieved by microwave in citrate buffer (pH 6), and thereafter sections were incubated in 0.3% H_2_O_2_/methanol for 30 min to block endogenous peroxidases, followed by incubation in phosphate buffered saline with 0.1% Tween 20 (PBST) containing 5% skim milk for 30 min to prevent nonspecific binding. Serial sections were then incubated with R58 (1 μg/ml) or L59 (1 μg/ml) overnight at 4°C. As negative controls, slides were incubated with nonimmune IgG under the same conditions. EnVision/HRP (DAKO) was used as the second antibody. Serial sections were also immunostained with anti-αSMA (diluted with 1:100, an activated HSC marker), anti-CD31 (5 μg/ml, a liver sinusoidal endothelial cell marker), and anti-F4/80 (5 μg/ml, a Kupffer cell marker) antibodies. The sections were counterstained with Hematoxylin. Fibrosis was detected using Sirius red as previously described (Junqueira et al. 1979). Both fibrotic areas and R58 positive areas were measured in each of three fields of five independent sections by WinROOF image analysis software.

### Immunofluorescent staining

Four μm of liver sections were deparaffinized and heated to 121°C in citrate buffer (pH6) by an autoclave for 10 min for antigen retrieval. After blocking with 3% BSA/10% normal goat serum/0.1 M glycine/PBST for 30 min, the sections were incubated with both anti-pSmad3C (2 μg/ml) and anti-αSMA (1:100) antibodies overnight at 4°C, followed by incubation with both Alexa Fluor 488 anti-rabbit IgG and Alexa Fluor 555 anti-mouse IgG (1:1000; red; Life Technologies, Carlsbad, CA) for 2 hours at room temperature. Thereafter, the sections were incubated with Hoechst 33258 (1:5000, DOJINDO Laboratories, Kumamoto, Japan) for 10 min at room temperature, and were mounted with Fluoromount (Diagnostic BioSystems, Pleasanton, CA). Image data were acquired on a Zeiss LSM 700 laser scanning confocal microscopy.

### Statistical analysis

Quantitative data are shown as mean ± SE. The two-sample Wilcoxon rank-sum test was employed to evaluate difference between two groups. A *p*-value < 0.01 was considered as statistically significant.

## Electronic supplementary material

Additional file 1: Figure S1: Sequence alignment of three pro-TGF-β isoforms. (PDF 596 KB)

Additional file 2: Figure S2: Isoform specificity of R58 antibody. (PDF 82 KB)

## Abbreviations

LAP: Latency-associated protein
TGF-β: Transforming growth factor-β
ECM: Extracellular matrix
SLC: Small latent complex
LTBP: Latent TGF-β binding protein
LLC: Large latent complex
MMP: Matrix metalloproteinase
PLK: Plasma kallikrein
LAP-DP: Degradation product of LAP
rhLAP β1: Recombinant human LAP β1
R^58^ LAP-DP: PLK-cleaved N-terminal side LAP-DP ending at R^58^
L^59^ LAP-DP: PLK-cleaved C-terminal side LAP-DP beginning from L59
GST: Glutathione-*S*-transferase
PLN: Plasmin
CCl4: Carbon tetrachloride
BDL: Bile duct ligation
αSMA: α-smooth muscle actin
HSC: Hepatic stellate cell
AIH: Autoimmune hepatitis
NASH: Non-alcoholic steatohepatitis
HE: Hematoxylin and Eosin
PBST: Phosphate buffered saline with 0.1% Tween 20.
