# Supplementary material for: LAP degradation product reflects plasma kallikrein-dependent TGF-β activation in patients with hepatic fibrosis
Source: Springerplus. 2014 May 1;3:221. doi: 10.1186/2193-1801-3-221 (PMC4033717; doi:10.1186/2193-1801-3-221)
Supplement: Supplementary file 1 — Additional file 1: Figure S1: Sequence alignment of three pro-TGF-β isoforms. (PDF 596 KB) [file 40064_2013_959_MOESM1_ESM.pdf]

Supplemental Data

Figure S1 Sequence alignment of three pro-TGF-β isoforms

Sequences of TGF-βs 1-3 are aligned (Roberts and Sporn 1990). Signal peptide portion is highlightened in light yellow. LAP portion is highlightened in light blue. Active TGF-β portion is highlightened in magenta. Asterisks indicate same amino acids among three isoforms. PLK cleavage site (R^{58}-L^{59}) is indicated by a red arrow. Synthetic peptides, having the sequences from I^{36} to S^{64} (TGF-β1), L^{27} to E^{55} (TGF-β2), L^{30} to E^{58} (TGF-β3) surrounded by a rectangle, were made for checking isoform-specificity of R58 antibody.

Supplemental Reference

Roberts AB, Sporn MB. (1990) The Transforming Growth Factor-βs, in Peptide Growth Factors and Their Receptors I. Sporn MB, Roberts AB (eds) Springer-Verlag. Berlin, Germany. 419-472.

```
                              signal peptide← →LAP                         ↓PLK
TGF-β1      MPPSGLRLLPLLLPLLWLLVLTPGRPAAGLSTCKTIDMELVKRKRIEAIRGQILSKLRLA  60
TGF-β2      --------MHYC-VLSAFLILHLVTVALSLSTCSTLDMDQFMRKRIEAIRGQILSKLKLT  51
TGF-β3      ------MKMHLQRALVVLALLNFATVSLSLSTCTTLDFGHIKKKRVEAIRGQILSKLRLT  54
                          *     *        **** * *      ** *********** *

TGF-β1      SPPSQGEVPPGPLPEAVLALYNSTRDRVAGESAEPEP-----EPEADYYAKEVTRVLMVE 115
TGF-β2      SPPEDYPEPEE-VPPEVISIYNSTRDLLQEKASRRAAACERERSDEEYYAKEVYKIDMPP 110
TGF-β3      SPPE-PTVMTH-VPYQVLALYNSTRELLEEMHGEREEGCTQENTESEYYAKEIHKFDMIQ 112
            ***          *  *   *****                      *****     *

TGF-β1      ---THNEIYDKFKQSTHSIYMFFNTSELREAVPEPVLLSRAELRLLRLK----LKVEQHV 168
TGF-β2      FFPSENAIPPTFYRPYFRIVR-FDVSAMEKNASN---LVKAEFRVFRLQNPKARVPEQRI 166
TGF-β3      GLAEHNELAVCPKGITSKVFR-FNVSSVEKNRTN---LFRAEFRVLRVPNPSSKRNEQRI 168
                 *                *  *           *  ** *  *         **

TGF-β1      ELYQKYSN-----NSWRYLSNRLLAPSDSPEWLSFDVTGVVRQWLSRGGEIEGFRLSAHC 223
TGF-β2      ELYQILKSKDLTSPTQRYIDSKVVKTRAEGEWLSFDVTDAVHEWLHHKDRNLGFKISLHC 226
TGF-β3      ELFQILRP-DEHIAKQRYIGGKNLPTRGTAEWLSFDVTDTVREWLLRESNLGLEISIHC 227
            ** *            **            ********  *  **       *   * **

TGF-β1      SC-----------DSRDNTLQVDINGFT---TGRRGDLATIH------GMNRPFLLLMAT 263
TGF-β2      PCCTFVPSNNYIIPNKSEELEARFAGIDGTSTYTSGDQKTIKSTRKKNSGKTPHLLLMLL 286
TGF-β3      PCHTFQP-NGDILENIHEVMEIKFKGVDNEDDHGRGDLGRLK---KQKDHHNPHLILMMI 283
             *                       *         **               * * **

                          LAP← →active TGF-β
TGF-β1      PLERAQHL--QSSRHRRALDTNYCFSSTEKNCCVRQLYIDFRKDLGWKWIHEPKGYHANF 321
TGF-β2      PSYRLESQ-QTNRRKKRALDAAYCFRNVQDNCCLRPLYIDFKRDLGWKWIHEPKGYNANF 345
TGF-β3      PPHRLDNPGQGGQRKKRALDTNYCFRNLEENCCVRPLYIDFRQDLGWKWVHEPKGYYANF 343
            *  *         *  ****  ***     *** * *****  ****** ****** ***

TGF-β1      CLGPCPYIWSLDTQYSKVLALYNQHNPGASAAPCCVPQALEPLPIVYYVGRKPKVEQLSN 381
TGF-β2      CAGACPYLWSSDTQHSRVLSLYNTINPEASASPCCVSQDLEPLTILYYIGKTPKIEQLSN 405
TGF-β3      CSGPCPYLRSADTTHSTVLGLYNTLNPEASASPCCVPQDLEPLTILYYVGRTPKVEQLSN 403
            * * ***  * **  * ** ***  ** *** **** * **** * ** *  ** *****

TGF-β1      MIVRSCKCS 390
TGF-β2      MIVKSCKCS 414
TGF-β3      MVVKSCKCS 412
            * * *****
```

Figure S1
