# Supplementary material for: LAP degradation product reflects plasma kallikrein-dependent TGF-β activation in patients with hepatic fibrosis
Source: Springerplus. 2014 May 1;3:221. doi: 10.1186/2193-1801-3-221 (PMC4033717; doi:10.1186/2193-1801-3-221)
Supplement: Supplementary file 2 — Additional file 2: Figure S2: Isoform specificity of R58 antibody. (PDF 82 KB) [file 40064_2013_959_MOESM2_ESM.pdf]

Supplemental Data

Figure S2 Isoform specificity of R58 antibody

Synthetic peptides derived from the indicated sequences of TGF-β1-3 LAP (refer to Figure S1) were incubated with/without PLK or PLN for 45 min at 37℃, and thereafter samples were spotted onto a nitrocellulose membrane followed by dot blot analyses using R58 antibody. *Lanes 1*, TGF-β1 LAP peptide; *lanes 2*, TGF-β2 LAP peptide; *lanes 3*, TGF-β3 LAP peptide. *Column 1*, undigested peptides; *column 2*, PLK-digested peptides; *column 3*, PLN-digested peptides. Representative results from three independent experiments with a similar result are shown.

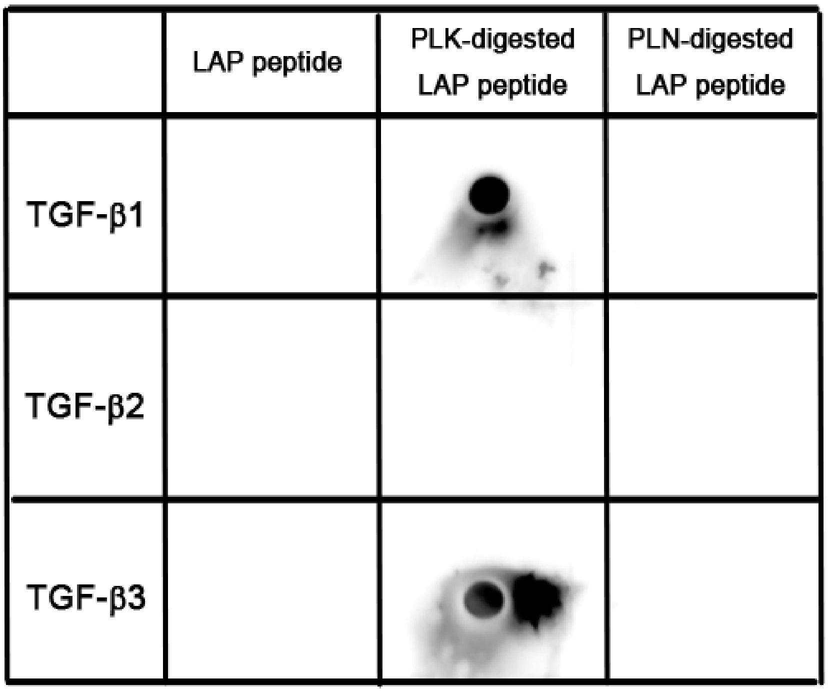

Figure S2
